# Enhanced genetic maps from family-based disease studies: population-specific comparisons

**DOI:** 10.1186/1471-2350-12-15

**Published:** 2011-01-19

**Authors:** Chunsheng He, Daniel E Weeks, Steven Buyske, Goncalo R Abecasis, William C Stewart, Tara C Matise

**Affiliations:** 1 Department of Genetics, Rutgers University, Piscataway, NJ, USA; 2 Laboratory of Statistical Genetics, Rockefeller University, New York, NY, USA; 3 Departments of Human Genetics and Biostatistics, Graduate School of Public Health, University of Pittsburgh, Pittsburgh, PA, USA; 4 Department of Statistics and Biostatistics, Rutgers University, Piscataway, NJ, USA; 5 Center for Statistical Genetics, University of Michigan, Ann Arbor, MI, USA; 6 Department of Biostatistics, Columbia University, New York, NY, USA; 7 See Acknowledgments for listing of members of the Enhanced Map Consortium

## Abstract

**Background:**

Accurate genetic maps are required for successful and efficient linkage mapping of disease genes. However, most available genome-wide genetic maps were built using only small collections of pedigrees, and therefore have large sampling errors. A large set of genetic studies genotyped by the NHLBI Mammalian Genotyping Service (MGS) provide appropriate data for generating more accurate maps.

**Results:**

We collected a large sample of uncleaned genotype data for 461 markers generated by the MGS using the Weber screening sets 9 and 10. This collection includes genotypes for over 4,400 pedigrees containing over 17,000 genotyped individuals from different populations. We identified and cleaned numerous relationship and genotyping errors, as well as verified the marker orders. We used this dataset to test for population-specific genetic maps, and to re-estimate the genetic map distances with greater precision; standard errors for all intervals are provided. The map-interval sizes from the European (or European descent), Chinese, and Hispanic samples are in quite good agreement with each other. We found one map interval on chromosome 8p with a statistically significant size difference between the European and Chinese samples, and several map intervals with significant size differences between the African American and Chinese samples. When comparing Palauan with European samples, a statistically significant difference was detected at the telomeric region of chromosome 11p. Several significant differences were also identified between populations in chromosomal and genome lengths.

**Conclusions:**

Our new population-specific screening set maps can be used to improve the accuracy of disease-mapping studies. As a result of the large sample size, the average length of the 95% confidence interval (CI) for a 10 cM map interval is only 2.4 cM, which is considerably smaller than on previously published maps.

## Background

Genetic maps are the foundation of linkage mapping for disease genes [1]. Accurate genetic maps can greatly increase the power of a linkage study, especially for multipoint analysis. The accuracy of genetic maps is largely a function of the number of actual recombination events present in the data. Despite the importance of precise genetic maps for linkage studies, most genome-wide genetic maps [2-7] were built using a small collection of pedigrees comprising only the eight largest families (188 meioses total) in the **C**entre d'**E**tude du **P**olymorphisme **H**umain (CEPH) reference panel [8]. Therefore, the 95% confidence interval (CI) for a 10 cM map interval from this small sample is large, at least 9.1 cM. deCODE Genetics constructed a substantially improved genetic map by genotyping 146 nuclear families containing 1,257 meioses [9,10]. However, primarily because the grandparents of these small families from Iceland were not genotyped, the average number of informative meioses is only approximately 400, leading to an average 95% CI of 6.1 cM for a 10 cM map interval.

Several studies have shown that the use of inaccurate genetic maps during linkage analysis can reduce power and induce bias in the results [11,12]. These effects are more pronounced for analyses using sex-specific maps, since they are each based on only half the meiotic count of sex-averaged maps, and therefore sampling errors pose an even greater problem. Halpern and Whittemore [13] showed that when distances from different maps were used in a multipoint analysis of prostate cancer, significantly different results can be produced.

We have used existing genotype data from 26 disease studies to generate improved genetic maps. The NHLBI Mammalian Genotyping Service (MGS) performed genome-wide linkage genotyping for hundreds of genetics studies using the Weber screening panels, with markers roughly evenly spaced along each chromosome at about 10 cM [14]. The genotypes that they generated for these studies are appropriate for the construction of more accurate maps. Here, we describe the construction of high precision sex-averaged and sex-specific genetic maps utilizing genotypes from over 4,000 pedigrees that were previously genotyped by the MGS. Constructing genetic maps on a large collection of general pedigrees is extremely computationally demanding, especially in the presence of genotype errors, missing data, and multiple ethnicities. We have effectively analyzed this large heterogeneous data collection, either by using joint analyses or by combining the results from individual datasets. Our datasets were derived from different self-described populations, such as European/European descent, Chinese, Hispanic, African American, and Palauan. There are suggestions that the distribution of recombination may vary among some populations [15-17]. Therefore, genotypes from different ethnic groups were also evaluated separately to test whether we could detect population-specific distributions of recombination, and to produce population-specific genetic maps for the populations for which we had sufficient data.

## Methods

### Data Collection

We sent requests for genotyping data to the PIs of 43 studies genotyped at the NHLBI MGS. We received fully de-identified genotype data for 26 datasets from 20 PIs (Table 1). These studies were genotyped using either Weber screening set 9 (387 markers, used in 15 studies) or screening set 10 (405 markers, used in 11 studies), which have 313 markers in common and an average marker heterozygosity of 0.74. Two additional PIs sent data for studies using Weber screening set 8 and 11. Since these were the only datasets that didn't use screening set 9 or 10 we opted to exclude them from our analyses. Overall, our data collection consisted of 4,461 pedigrees with 17,871 genotyped individuals. The pedigree structures included sibships, small nuclear families, and large extended pedigrees. While the vast majority of the pedigrees were small, there were also some very large pedigrees. The pedigree sizes ranged from 3 to 239 individuals per family, with a mean of 6.1 and a median of 4.

**Table 1:** A list of projects that contributed data^1^

PI (last name)	Number of persons genotyped	Number of pedigrees	Number of markers	Weber Set	Ethnicity	PubMed ID
Bell	602	60	386	9	European (Europe)	14500288
Berrettini	689	202	386	9	European (Europe)	11799475
Berrettini	899	329	361	9	European (USA^2^)	11799475
Boomsma	917	219	405	10	European (Europe)	17700629, 16899170
Cantor	347	71	402	10	European (USA)	15476245
Catalona	513	232	387	9	European (USA)	11309685
Cho	701	196	387	9	European (USA)	15472510
Concannon	465	111	387	9	European (USA)	11507694
Concannon	461	110	386	9	European (USA)	11507694
DeLisi	446	114	404	10	Hispanic (Costa Rica)	12116183
Duerr	439	96	386	9	European (USA)	10747815
Duerr	605	126	386	9	European (USA)	10747815
Hunt	3322	948	391	9	African American	12068377
Jacob	1041	375	402	10	European (Europe)	11818963
Jacob	550	191	402	10	European (Europe)	11818963
Klein	542	96	404	10	European (USA)	12900797
Klein	130	22	386	9	European (USA)	12900797
Kuivaniemi	140	38	402	10	European (Europe, USA, Canada)	15096456
Leal	153	11	386	9	European (Europe)	10777717
Murray	756	173	387	9	European (Europe, USA), Chinese	12087515
Myles- Worsley	275	18	404	10	Palauan	15915326
Pirastu	877	197	403	10	European (Europe)	15478097
Vats	220	23	404	10	Unknown^3^	12819239
Weiss	1128	279	404	10	Chinese	11673820
Xu	745	168	388	9	Chinese	10330357
Xu	749	172	387	9	Chinese	10330357

^1 ^Two additional PIs, A. Shuldiner and and G. Tromp, sent data for studies using Weber screening set 8 and 11. Since these were the only datasets that didn't use screening set 9 or 10 we opted to exclude them from our analyses.

^2 ^These samples are individuals of European descent living in the United States (USA).

^3 ^The ethnicity for the samples in this set was not available. Therefore, this set was only used for the initial CRI-MAP map which was used to determine starting points for the full-likelihood map.

The subjects were primarily Europeans or Americans of European descent (referred to throughout as Europeans), but several other self-described ethnic groups, such as Chinese, Hispanic, African American, and Palauan, were also represented in the data. The data from Chinese and African American populations included thousands of individuals. A Hispanic population was genotyped in a large dataset from Costa Rica. We also obtained a unique sample from the isolated Pacific island of Palau. The sample sizes in these populations are quite large (Table 2; data in this table are after thorough data cleaning) and are suitable for population-specific map construction and between-group map comparisons. Several of the study sets also included individuals from other populations but these samples sizes were too small to include in our analyses.

**Table 2:** Summary of our cleaned data in five populations

Population	Weber Sets	Number of Pedigrees	Number of Genotyped Individuals
European^1^	9, 10	2,521	9,380
Chinese	9, 10	653	2,830
African-American	9	942	2,594
Hispanic	10	97	408
Palauan	10	24	313

Total		4,237	15,525

^1 ^"European" also includes Americans of European descent.

### Data Cleaning

For rigorous quality control, we requested uncleaned genotype data and corresponding family relationship information from the PIs, and we performed thorough data cleaning. While we requested uncleaned genotype data so that we could apply an identical cleaning protocol to all the data sets, the primary studies of these data applied their own rigorous data cleaning steps prior to their own analyses. We evaluated the amount of missing genotype data per study and per marker to help ensure that none of the studies or markers were especially poorly genotyped. We identified and cleaned pedigree relationship errors first, followed by genotyping and gender-assignment errors. Pedigree relationship errors can result from different sources, such as undisclosed adoptions, mis-paternity, sample mix-up, incorrect family history, among others, all of which can lead to inaccurate results in linkage analyses. Genome scan data can be highly informative for checking the pedigree errors. We employed PREST [18] in our study, which implements identity-by-state, identity-by-descent, and likelihood-based methods to test whether the pattern of allele sharing between relative pairs is consistent with the stated relationship in the pedigrees. Individuals whose relationships in a pedigree were clearly wrong were excluded from our map construction. Genotyping errors can dramatically reduce the power of linkage studies. We used PedCheck [19] to identify, and clean our data of, Mendelian inconsistencies. For each marker that shows Mendelian inconsistencies, the PedCheck cleaning function sets all genotypes to unknown for each pedigree with inconsistencies. We detected subjects that were assigned an incorrect gender through identification of an over-abundance of homozygous female or heterozygous male genotypes for markers on the X chromosome. We identified 124 individuals that were coded as males that were highly likely to be females, or vice versa. The data included 6 markers from the Y chromosome non-pseudoautosomal region. Since males should be homozygous for these Y markers, any heterozygous Y genotypes suggest genotyping errors. Therefore, these markers provide genotyping error rate information. In summary, we detected 11 heterozygous genotypes in 35,375 Y genotypes (0.016%).

### Handling large pedigrees

Several linkage programs based on the Lander-Green algorithms have been developed, each with specific advantages and disadvantages. We are not aware of any single program that could perform all of the types of analyses required for our study, so we employed a combination of five programs: Allegro [20], CRI-MAP [21], MENDEL[22], MERLIN [23], and METAMAP [24]. While the vast majority of our pedigrees were small enough for Allegro and MERLIN to handle, we had some very large pedigrees that had to be split into smaller sub-pedigrees. We either split or trimmed our large pedigrees (N = 80) into smaller sub-pedigrees for analyses with Allegro and Merlin; this was not necessary for analyses with CRI-MAP. This trimming and splitting reduced our computational time by more than 93%. To construct a single, accurate estimate of the map based on data from two or more populations, we used the program METAMAP to combine the population-specific map estimates.

### Study- and Population-specific Marker Alleles

Even though all the genotyping was performed in the same center, the codes used by PIs to describe marker alleles are not necessarily consistent across all studies. To handle this problem, we obtained PCR bandsizes rather than allele codes from each PI. In some rare cases MGS devised multiple primers for the same marker and the allele sizes changed when the primers were altered. Therefore, it was important to use study-specific marker allele labels and allele frequencies when different primers were used in different studies for the same marker. In addition, since different populations might have different allele frequencies, population-specific alleles were also required. We incorporated study- and population-specific marker alleles into our linkage analyses by creating study/population-specific marker copies or by adjusting the PCR bandsizes to be the same for different primers.

### Ordering Markers and Comparing with the Physical Maps

Determining the correct marker orders was the first step in our map construction. Discrepancies have been previously noted between some of the Weber screening sets and physical positions [25]. We used the Marshfield genetic maps [7] and Weber screening set maps [14] to initially determine marker order. Physical positions of the markers were obtained from NCBI and UCSC Human Genome Browser. We used Multithreaded Electronic PCR (me-PCR) [26,27] to identify physical positions for markers not already identified in the published sequence. When comparing the marker order from the published maps with order determined on assembled sequence, we also identified a few discrepancies with the screening set map orders. We used linkage analysis of our data to resolve these marker order discrepancies and determine the final map order. In all cases, the linkage analyses we performed confirmed the physical order.

### Precise Estimation of Map Distances

With markers carefully ordered, we computed accurate map distances. We also tested the hypothesis that the distribution of recombination does not vary significantly among different ethnic groups. CRI-MAP is the only program that could handle all of our large pedigrees intact and it runs very quickly. Therefore, we used CRI-MAP for initial estimates of inter-marker distances. However, because CRI-MAP does not perform full-likelihood analyses, some level of information loss is expected, which can lead to potential biases in parameter estimates [28,29]. Therefore, we calculated more accurate map distance estimates by using the full-likelihood program, Allegro. Allegro applies the expectation-maximization (EM) algorithm [30] for map estimation and can be used for estimation of both sex-averaged and sex-specific maps. Because our European data set was extremely large and was derived from different marker sets, we first built maps separately for each Weber set (9 and 10) and then combined them together using the METAMAP program [24]; we did the same for the Chinese data set. METAMAP combines maps from different Weber sets (i.e. different studies) using weights that are inversely proportional to the variance of map distance estimates. The variances used by METAMAP were estimated using the non-parametric bootstrap [31].

### Testing for Population-specific Recombination

We used numerical optimization with the MERLIN program to compute the map distances and corresponding variance-covariance matrices for the data in each population. MERLIN does not currently have any built-in map estimation routines. However, it can compute the log-likelihood of the pedigree data for a given map. In order to estimate our map distance, we used the box-constrained optimization function "optim" of the R programming environment (L-BFGS-B method;[32]) to maximize the log-likelihood. The "optim" function optionally returns the Hessian matrix at the convergence point. Inverting the Hessian produced the variance-covariance matrix, which we used in the Wald test for statistical comparisons of the population-specific genetic maps. The variance of chromosomal and genome length was obtained by summing the individual terms in the variance-covariance matrix. We evaluated whether there are any differences between the maps, and if so, where the differences lie. We compared pairs of maps to identify differences in the estimated size of a) individual map intervals, b) individual chromosome map lengths, and c) map length over the entire genome. When performing multiple statistical tests, the Type 1 error rate may increase considerably. Using the QVALUE program [33], we corrected the multiple comparisons at a genome-wide level by controlling the Benjamini-Hochberg False Discovery Rate (FDR)[34]. We presented the p-values after correction for multiple testing. A significance level of 0.05 was used in all the tests.

## Results

### Data Cleaning

Among the 26 studies used in our analyses, the median amount of missing genotype data was 3.3% with only two studies missing more than 10% of genotypes (13% and 15%, respectively). Only 0.9% of the markers were missing more than 20% of genotypes, with most having a high missing rate in only a single study and none having a high missing data rate in more than three studies. Many pedigree errors were identified in the uncleaned data that we received. Problems that frequently occurred included half siblings coded as full siblings, non-biological sibs coded as biological sibs, and non-biological children present in the pedigrees. In some rare cases, more complex relationship mistakes were identified. We detected incorrect familial relationships in 129 families. We corrected 75 of them by deleting 124 problematic pedigree members, and removed the remaining 54 entire families that had serious relationship errors. In total, we deleted 499 individuals that accounted for about 3% of the data to eliminate these pedigree errors. Additional pedigrees were excluded from analysis if they did not match one of the 5 main ethnic populations or if pedigree-relationship data were not provided. Next, PedCheck detected approximately 10,000 Mendelian inconsistencies and 1.8% of the genotypes in our study were removed by PedCheck to create Mendelianly-consistent data.

Our final cleaned data contained 15,525 genotyped individuals from 4,237 pedigrees with 5.7 million genotypes (Table 2). The accuracy of map estimates relies greatly on the sample size. The improvement of map distance estimates as the sample size increased was evident. CRI-MAP detected an average of 7,926 informative meioses for our markers. Using 7,926 informative meioses, the expected 95% CI [35] of 10 cM is 1.6 cM, which is much smaller than on any existing maps.

### Marker Orders

We determined the map order for the markers used in these disease-mapping studies. Most of the markers are present on the Marshfield map. While the map orders on the Marshfield map, Weber set maps, and physical maps were consistent with each other for the majority of the markers, we found several mistakes in the Marshfield map and the Weber screenset maps. Linkage results from CRI-MAP were used to clarify these map order problems. Marker D20S159 was assigned to chromosome 20 on the Marshfield and Weber set 10 maps. However, both its physical location and our linkage results confirmed that it is located on chromosome 2. Also, an X chromosome marker, DXS9893, was assigned to an incorrect position in the Weber set 10, where it was listed as being about 44 cM upstream of the position identified by me-PCR and confirmed by our linkage analysis. We also detected two minor map order inversions in the Marshfield map, one on chromosome 6 (the correct order: D6S1034-D6S1006-D6S2434) and another on chromosome 20 (the correct order: D20S451-D20S164-D20S171). Linkage analyses confirmed that the physical map orders are correct for both of these cases.

### Enhanced Genetic Maps

The majority of our data were from Europeans (Table 2), providing a single-population sample size large enough to build genetic maps at high precision. Sex-specific and sex-averaged recombination rates in the European data were estimated with Allegro, using starting values as estimated by CRI-MAP. Recombination fractions were converted to genetic distances using the Kosambi map function so that they were directly comparable with the Marshfield map. Our enhanced genetic map contains 461 markers genotyped in Weber sets 9 and 10. The sex-averaged, female, and male maps had a total length of 3,741 cM, 4,762 cM, and 2,801 cM, respectively (Table 3). The female:male map length ratio, which ranged from 1.26 (chromosome 21) to 1.85 (chromosome 8), averaged 1.64 across all the autosomes. The largest inter-marker spacings were 22.6 cM, 30.7 cM, and 25.5 cM for the sex-averaged, female and male maps, respectively. Overall, our maps are about 7% longer than the Marshfield map.

**Table 3:** Genetic map lengths in different populations (Kosambi cM)

Chromosome	European Weber Set 9 and 10			Chinese Weber Set 9 and 10			African-American Weber Set 9			Hispanic Weber Set 10			Palauan Weber Set 10		
					
	Averaged	Female	Male	Averaged	Female	Male	Averaged	Female	Male	Averaged	Female	Male	Averaged	Female	Male
1	284.8	371.5	214.6	270.9	352.3	213.4	289.8	396.9	169.8	257.1	317.0	206.6	266.2	369.1	176.8
2	274.6	348.5	218.0	264.5	326.6	229.9	285.8	375.9	175.7	270.8	332.7	219.2	267.6	376.1	186.7
3	233.0	299.8	182.5	226.7	287.2	190.1	240.7	319.8	148.0	234.6	293.2	190.5	224.4	324.2	153.3
4	216.4	286.4	162.2	204.1	278.5	153.1	211.7	290.7	122.3	212.2	256.6	174.3	208.4	272.4	146.1
5	213.0	280.4	160.0	206.6	271.7	164.7	212.8	296.9	122.5	201.1	242.9	167.8	199.3	277.0	143.1
6	197.5	259.4	153.0	195.5	261.3	157.6	190.6	266.2	107.1	185.0	257.3	129.4	187.4	290.2	114.7
7	190.9	246.7	145.6	185.0	238.2	147.4	186.8	250.6	111.4	182.6	208.1	159.7	193.1	285.7	121.9
8	171.5	231.8	125.4	170.8	228.0	130.7	176.5	242.6	102.9	156.2	221.8	101.0	165.2	213.7	119.9
9	168.5	208.8	139.0	172.3	213.0	145.5	155.5	214.1	94.7	165.3	201.0	137.8	188.1	248.5	137.1
10	175.5	236.2	131.4	174.8	226.5	141.8	180.3	243.9	108.9	174.7	230.3	142.2	187.8	247.5	138.0
11	165.9	217.9	128.3	156.6	201.0	132.1	166.8	224.3	106.2	153.0	191.0	123.7	147.5	178.2	116.1
12	168.1	218.5	128.7	160.9	214.0	124.9	168.4	225.7	102.1	158.6	191.2	133.6	147.8	208.4	98.4
13	124.1	157.9	99.5	118.9	156.2	96.3	128.2	163.4	80.2	121.0	146.8	95.6	139.9	181.6	106.8
14	124.9	150.0	101.6	110.3	128.7	95.6	117.6	150.9	73.0	131.8	151.4	113.0	113.9	141.0	86.9
15	127.0	152.6	110.0	126.2	155.5	112.3	122.3	150.3	81.1	122.7	150.5	101.0	134.7	179.9	105.7
16	133.5	166.9	109.2	128.9	160.6	116.3	127.0	175.3	71.8	132.1	164.6	106.0	135.5	171.4	108.2
17	138.8	174.2	115.3	135.4	163.6	126.5	147.6	184.9	97.6	138.7	174.8	106.6	159.2	191.2	119.5
18	121.3	157.3	95.1	112.5	139.2	91.9	121.8	165.7	72.0	113.6	136.0	97.3	121.3	151.9	91.5
19	102.6	133.0	85.5	101.5	124.3	94.1	107.9	135.2	72.8	93.9	107.4	85.0	94.2	115.3	80.4
20	102.6	130.3	82.0	103.8	129.5	89.5	106.4	140.8	62.0	94.3	118.6	76.9	110.5	145.1	87.1
21	63.7	72.5	57.7	63.0	70.4	60.2	72.3	94.8	42.3	58.7	63.3	54.9	62.4	71.5	53.9
22	69.5	88.5	56.7	75.4	96.5	60.6	46.7	66.8	25.3	76.6	89.0	65.3	68.9	75.9	60.6
X	172.9	172.9		158.2	158.2		165.0	165.0		142.2	142.2		177.3	177.3	
				
Total (Kosambi)	3740.5	4761.9	2801.2	3622.9	4581.0	2874.2	3728.5	4940.5	2149.6	3576.7	4387.7	2787.3	3700.6	4892.9	2552.7
				
Total (Haldane)	4130.8	5424.0	3063.2	3997.0	5212.4	3155.2	4182.4	5752.5	2324.9	3985.2	5048.4	3101.2	4160.1	5743.2	2832.7

Note: the map lengths in this table were computed using all markers genotyped in each population. These lengths are different than the lengths we estimated for the between-population comparisons (Table 4), where only markers common to each pair of populations were used to estimate map lengths. The European and Chinese maps included 438 map intervals, while the set African-American maps had 351 intervals and the Hispanic and Palauan maps had 369 intervals.

Due to the large sample size, we observed a large number of informative meioses, which statistically ensured the accuracy of our map estimates. The standard error of a 10 cM map interval was only 0.6 cM on the sex-averaged map. Therefore, for a map interval of 10 cM, the estimated 95% CI was only 2.4 cM long. Since only about half the meioses were used when estimating each sex-specific map, the standard errors that we observed in female and male maps were a bit larger than those in the sex-averaged maps: for a map interval of 10 cM, the sex-specific standard errors were usually around 1 cM.

Our detailed sex-averaged maps, female maps, male maps, and their corresponding standard error (S.E. for theta) in each map interval are listed in Additional File 1: European_maps.xls. Population-specific map distances were estimated using the African American, Chinese, Hispanic and Palauan datasets and are described below (see "*Genetic maps from non-European Populations" *and Additional Files 2, 3, 4, 5: Chinese_maps.xls, African American_maps.xls, Hispanic_maps.xls, Palauan_maps.xls.)

### Null Alleles

In response to a concern raised during the review process about the possible impact of null alleles on our results, we evaluated the frequency of null alleles in our data [16]. We used the MENDEL program to estimate the null allele frequency of each marker, separately by population. We found only one marker had a null allele frequency > 0.05, a level below which our simulations indicate negligible impact of null alleles on the accuracy of estimates of recombination rate (data not shown). This marker was D6S1959, which Jorgenson et al. [16] also found to have a null allele, and its frequency was > 0.05 in the African American, Chinese, and Palauan datasets. As a sensitivity analysis, we compared the map lengths obtained ignoring null alleles for the two map intervals flanking this marker with estimates obtained while modeling null alleles using MENDEL. In all three populations where the null allele frequency is > 0.05, the estimates of recombination fraction allowing for null alleles are very similar to the estimates obtained with a conventional analysis that does not allow for null alleles. Therefore, with only one marker out of 461 showing a modest frequency of null alleles, and having demonstrated that the impact of that marker on our map estimates is small, we are confident that null alleles do not have a substantial impact on our analyses and conclusions.

### Comparing Our Maps with Marshfield/Weber Maps

**Figure 1:**
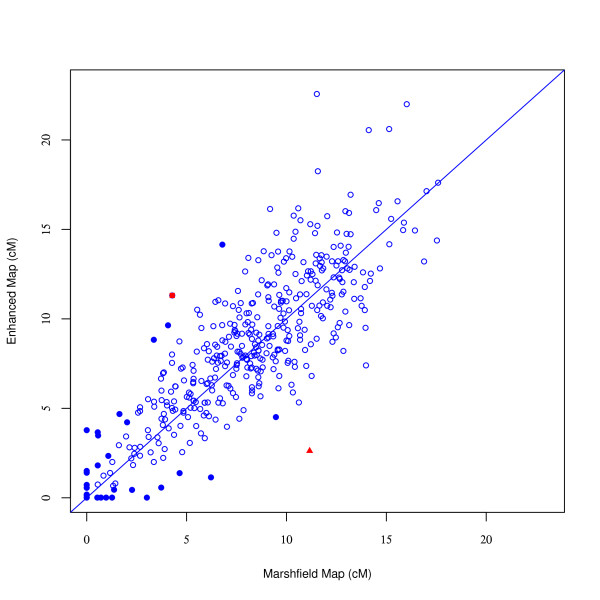
**Comparing map interval lengths between the Marshfield map and our enhanced map (Kosambi cM)**. The solid red triangle indicates a map interval on chromosome 20 with incorrect Marshfield map order (D20S451-D20S164: 11.16 cM in Marshfield map vs. 2.62 cM in our European map). The solid red square represents a map interval on chromosome 11 (D11S1999-D11S1981: 4.28 cM in Marshfield map vs. 11.31 cM in our map). Other map intervals exhibiting over two-fold difference in length are depicted with solid blue circles.

The Weber screening sets were derived from the Marshfield map, and therefore we compared our map distance with the Marshfield map. Figure 1 shows many map intervals with large differences in map length. For example, at the map interval D20S451-D20S164 on chromosome 20 (depicted with a solid triangle), the Marshfield map has a map distance of 11.16 cM, while our map showed a map distance of 2.62 cM. This map interval has such a high length discrepancy because the order of these markers was different (incorrect) on the Marshfield map. Another map interval D11S1999-D11S1981 on chromosome 11 (depicted in a solid square) had a Marshfield map length of 4.28 cM, while our map showed a length of 11.31 cM. In this example the order of markers is consistent between our map and the Marshfield map. Use of imprecise map distances can impact the accuracy of multi-point linkage results. When different genetic map distances are used for the same linkage study, different conclusions could be reached. Since many map intervals differ greatly between the Marshfield map and our enhanced map, investigators who use Weber screensets should obtain more accurate linkage results by using our enhanced maps.

### Comparisons of Population-specific Maps

**Figure 2:**
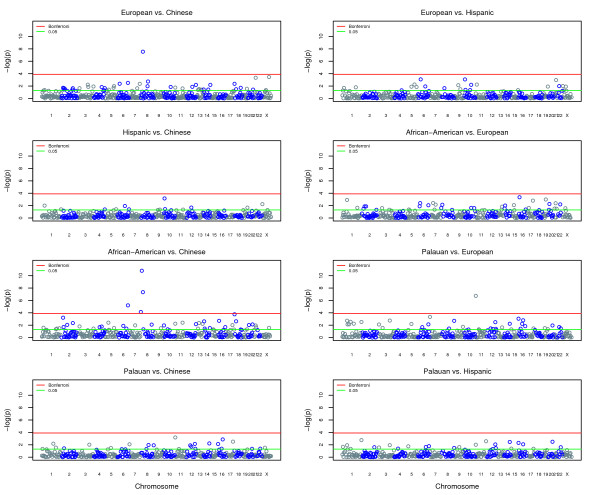
**Population-specific comparisons of map interval lengths**. The - log_10_(p-values) measuring map interval differences are plotted in chromosomal order, with chromosomes shown in alternating colors for clarity. The green line indicates the p = 0.05 significance threshold while the red line indicates the Bonferroni-corrected significance threshold.

**Figure 3:**
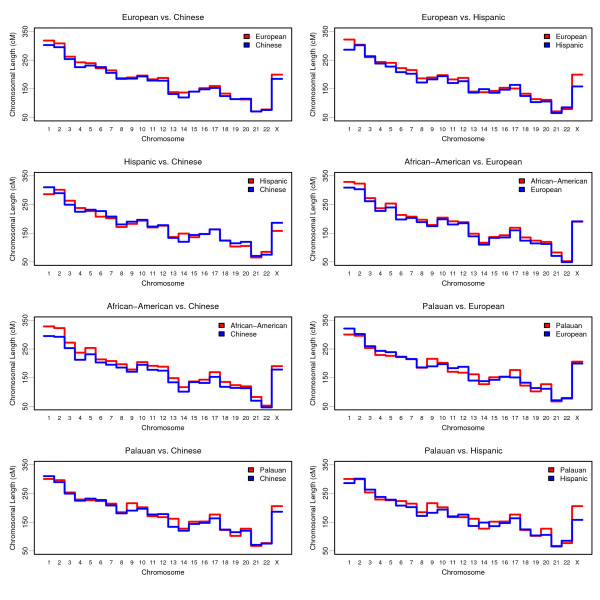
**Chromosomal length comparisons between populations**. The chromosomal lengths are depicted in different colors (red vs. blue) as shown in the legend. The chromosomal length is expressed in Haldane cM on the Y-axis, while the X-axis indicates chromosomes.

We were able to perform eight population-specific comparisons, comparing map distance estimates in populations genotyped for the same screening sets (Table 4). The between-group comparisons are shown as Manhattan plots in Figure 2 and Q-Q plots in Additional File 6: Q-Q_plot.pdf. The chromosomal length comparisons are also illustrated in Figure 3. Since studies from the same screenset might have slight differences in the markers actually used, for each comparison, we compared maps constructed *de novo *using only the markers shared between the two groups. Therefore, since slightly different sets of markers are used, the resulting map lengths are specific to each analysis. For example, the Chinese map length when being compared to the European map is 4,036 cM, while it is 4,063 cM when compared to the Hispanic map. The Merlin program used for these analyses uses the Haldane map function, so our map lengths from these population comparisons are not directly comparable with Kosambi map lengths described elsewhere in this paper.

**Table 4:** Summary of significant between-population map comparison results

Samples Compared	Number of Intervals	Weber Sets	Significant sex-averaged Intervals	Significant Chromosomes	Overall sex-averaged length (Haldane cM)
European vs. Chinese	441	9 and 10	chromosome 8p: D8S1130-D8S1106 (p = 1.17E-05)	4, 14	p = 7.29E-8 (4,185 vs. 4,036 cM)

European vs. Hispanic	370	10	NS	1, X	p = 2.96E-5 (4,183 vs. 3997 cM)

Chinese vs. Hispanic	370	10	NS	14	NS *(p = 0.19)* (4,063 vs. 3,997 cM)

African-American vs. European	358	9	NS	1, 2, 5, 6, 11, 18, 19, 21	p < 1E-10 (4,218 vs. 4,011 cM)

African-American vs. Chinese	358	9	chromosome 2p: D2S2952-D2S1400 (p = 0.036) chromosome 6q: D6S305-D6S1277 (p = 0.0008) chromosome 8p: D8S264-D8S277 (p = 0.0065) chromosome 8p: D8S277-D8S1130 (p = 5.80E-09) chromosome 8p: D8S1130-D8S1106 (p = 8.29E-06) chromosome 18p: GATA178F11-D18S481 (p = 0.012)	1, 2, 3, 4, 5, 7, 11, 12, 13, 14, 16, 17, 18, 21	p < 1E-10 (4,218 vs. 3,883 cM)

European vs. Palauan	368	10	chromosome 11p: D11S1984-D11S2362 (p = 6.8E-05)	NS	NS *(p = 0.70)* (4,182 vs. 4,158 cM)

Chinese vs. Palauan	368	10	NS	NS	NS *(p = 0.17)* (4,066 vs. 4,158 cM)

Hispanic vs. Palauan	369	10	NS	NS	p = 0.03 (3,998 vs.4,158 cM)

Note: NS stands for "no significant findings"

Several significant differences were identified when comparing the European and Chinese data. The European map was significantly longer than the Chinese map (European = 4,185 cM, Chinese = 4,036 cM, p = 7.29E-08); most chromosome maps were longer with chromosomes 4 and 14 being statistically significant (p = 0.04 and p = 1.47E-04, respectively); and one specific map interval was significantly longer in the European map (5.3 cM) compared with the Chinese map (1.7 cM): 8p: D8S1130-D8S1106 (p = 1.17E-05) (Table 4). An 8p inversion polymorphism has been previously reported at this map location[36].

We did not observe any significant map interval differences between the Hispanic and European maps after the correction for multiple testing. However, two chromosomes, chromosome 1 (p = 0.01) and the X chromosome (p = 0.01), showed significant differences in length. The chromosome 1 and X maps were 12% and 26% longer in Europeans than in Hispanics, respectively. The overall map length was also significantly different (European = 4,183 cM, Hispanic = 3,997 cM, p = 2.96E-05), with the European map about 5% longer than the Hispanic map (Table 4).

The Chinese and Hispanic samples were also compared with each other using the Weber set 10 markers. The smallest p-value was observed at the map interval D10S189-D10S1412 on chromosome 10, but it is not genome-wide significant (p = 0.26). For the map length comparisons, the difference was significant only for chromosome 14 (p = 0.04), where the Hispanic map was about 23% longer than the Chinese (Table 4). The overall Hispanic map was about 2% shorter than the Chinese map, which is not significant (Chinese = 4,063 cM, Hispanic = 3,997 cM, p = 0.19).

African American data were genotyped using the Weber set 9. When comparing it with the European data, we did not observe any map interval differences of genome-wide significance after the correction for multiple testing. The smallest p-value is only 0.14 which was located at the map interval D16S748-D16S764 on chromosome 16. The African American data had longer map lengths for all the autosomes, whereas the map length for the X chromosome was nearly the same. The differences are significant for eight chromosomes (1, 2, 5, 6, 11, 18, 19, and 21). Finally, the difference in the overall map length was highly significant (African American = 4,218 cM, European = 4,011 cM, p < 1E-10) between the two populations, with the African American map about 5% longer than the European map (Table 4).

We also compared the African American population with the Chinese population using the Weber set 9, and we detected 6 map interval differences of genome-wide significance. The p-values were very small. For these four map intervals, one was located on chromosome 6 (D6S305-D6S1277) and the three others were consecutively located on chromosome 8 short arm (D8S264-D8S277-D8S1130-D8S1106). The D6S305-D6S1277 interval size was 7.3 cM and 3.2 cM in the African American and Chinese data, respectively. The three consecutive map intervals on chromosome 8p were located in the same common inversion polymorphism region as we observed between the European and Chinese maps. The African American map interval sizes were 17.5 cM, 4.4 cM, and 6.0 cM, while the Chinese sizes were 10.8 cM, 12.9 cM, and 1.8 cM, respectively. The other two significant map intervals at FDR = 0.05 level were located on chromosome 2 (D2S2952-D2S1400) and on chromosome 18 (GATA178F11-D18S481) with p = 0.036 and p = 0.012, respectively. The African American data have longer map lengths for all the chromosomes and differences are significant for 14 of them. The largest difference was observed at chromosome 21 where the African American map was about 19% longer than the Chinese map. The overall African American map was 9% longer than the Chinese map, and this difference was highly statistically significant (African American = 4,218 cM, Chinese = 3,883 cM, p < 1E-10) (Table 4).

We also compared the Palauan and European data using the Weber set 10 markers. One map interval difference of genome-wide significance, D11S1984-D11S2362 (p = 6.8E-05), was at the distal end of chromosome 11 short arm (Table 4). The map distance in the Palauan and European data was 2.1 cM and 9.5 cM, respectively. The map lengths of each chromosome and the overall map length in the two populations did not differ significantly (European = 4,182 cM, Palauan = 4,158 cM, p = 0.70). When the Palauan data were compared with the Chinese data, we observed the smallest p-value at the same D11S1984-D11S2362 map interval, which, however, is not statistically significant (p = 0.24). We did not observe any significant difference in the overall map lengths (Chinese = 4,066 cM, Palauan = 4,158 cM, p = 0.17) or chromosome map length, either. When the Palauan results was compared with the Hispanic results, the only significant difference detected is the overall map length (Hispanic = 3,998 cM, Palauan = 4,158 cM, p = 0.03), where the Palauan map was about 4% longer than the Hispanic map (Table 4).

### Genetic maps from non-European Populations

Because we observed significant map-length differences between some population groups, we also separately constructed sex-averaged and sex-specific genetic maps in the four non-European populations using Allegro. These maps are summarized in Table 3 and are included as Additional Files 2, 3, 4, 5 (Chinese_maps.xls, African American_maps.xls, Hispanic_maps.xls, Palauan_maps.xls). The Chinese and African American sample sizes are large, so their data alone can provide accurate map estimates for future linkage scans in the two populations. Our Hispanic and Palauan sample sizes are comparatively small. Map lengths for each of these populations were estimated using different sets of markers, so their map lengths are not directly comparable with each other.

## Discussion

We have constructed high-precision genetic maps with a very large data set generated by the NHLBI Mammalian Genotyping Service (MGS) and performed a systematic comparison of genetic maps across different populations. Accurate gene mapping requires high quality genetic maps. However, errors from a variety of sources cannot be avoided. We collected the genotype data in an uncleaned format and performed thorough and consistent data cleaning. By using the program PREST, we verified pedigree structures and over one hundred pedigrees with relationship errors were detected in these samples. Data with undetected pedigree errors could lead to inaccurate linkage results that can influence the conclusion regarding the presence or the absence of a linkage [37]. The fact that we found so many relationship errors in these uncleaned data is a reminder of the need for a rigorous verification of pedigree information in linkage studies.

Different studies may use different labels to represent alleles and allele frequencies may vary in different populations. Therefore, it is important for linkage programs to use study/population-specific marker allele labels and frequencies when jointly analyzing data from different studies and populations. Unfortunately, most available linkage software (except newer versions of MENDEL [38]) cannot handle study/population-specific alleles directly. In this study, we employed two very useful approaches: we created dummy marker copies in each dataset for any markers genotyped in different studies or made proper bandsize adjustment for those markers that were genotyped by multiple primers. We were then able implement linkage analyses using study/population-specific alleles without the need to modify existing software.

By comparing the genetic orders of autosomal markers from the Weber sets 9 and 10 with their physical positions, DeWan et al. [25] identified 7 markers in the Set 10 and 5 markers in Set 9 whose physical orders were inconsistent with their genetic orders. With our large data collection, we confirmed that most of these previously-identified inconsistencies resulted from the imprecision of the physical map used in that comparison. With the latest (more accurate) physical assembly data, we only detected one inconsistency that had been encountered by DeWan et al: marker D20S159 was assigned to the wrong chromosome in the Weber set 10 (assigned to chromosome 20 instead of chromosome 2). In addition, we identified a marker order mistake on the X chromosome: marker DXS9893 in the Weber set 10 was incorrectly placed position 44 cM upstream of its actual location. These ordering problems could seriously impair the validity and accuracy of results of any linkage analysis that used these markers. In order to obtain correct linkage results for previously published genome scans, multipoint linkage analyses should be repeated on these regions with the correct map orders.

We tested population-specific recombinations across five ethnic groups. Numerical optimization is extremely time-consuming when the number of estimated recombination fractions (*N*) becomes large because the computational complexity is generally on the order of *N^2 ^*for each iteration. The great advantage of the numerical optimization method is that we can incorporate the covariance terms into our calculation as well as directly confirm the success of convergence, which improves our statistical tests. It was necessary to include the covariance terms because the map distance estimates of map intervals on the same chromosome are not always independent. Our results showed that adjacent map interval estimates are usually negatively correlated with each other, while the map intervals far apart tend to be independent (results not shown).

When comparing the maps interval by interval, the results from the European, Chinese, and Hispanic samples were in quite good agreement with each other. One region on chromosome 8p showed significant length differences between the European and Chinese maps, and between the African American and Chinese maps. This map interval lies within the 8p (8p23.1-8p22) inversion polymorphism region [36], which also harbors recurrent chromosomal rearrangements, including an inverted duplication deletion (8p23) [17,39,40]. This region harbors several members of the olfactory gene receptor family and is flanked by repeated inverted sequences which mediate homologous unequal recombination [39]. The frequency of 8p inversion carriers has been estimated at 39% in a Japanese population and 26% in Europeans [39,40]. Since an inversion has the potential to influence the computed map distance, either by suppressing recombination or altering regional physical distances, different map lengths could be observed when inversion frequencies differ among populations. Due to the sparseness of the Weber screening sets, it is not possible for us to investigate the potential impact of this inversion polymorphism on these maps in more detail. We also detected three other significantly different map intervals when comparing the African American and the Chinese samples (Table 4).

A highly significant difference in the D11S1984-D11S2362 map interval size was observed between the Palauans and the Europeans. This map interval is located within 5 Mb of the beginning of chromosome 11, where an exceptionally high level of structural variations have been reported recently. Tuzun et al. [41] identified 297 sites of structural variations (inversions, deletions, and insertions) in the whole genome, six of which were clustered in this narrow region. It would be interesting to evaluate the Palau-island population for the presence and frequency of structural variants in this region.

We also compared the map lengths of individual chromosomes and of the entire genome across the populations. We identified several chromosomes with significantly different map lengths between populations, and the full-genome-length comparisons showed the African American map to be longer than the European and Chinese maps (consistent with Jorgenson et al. [16]), the European map to be longer than the Chinese map (consistent with Ju et al. [17]), and the European map to be longer than the Hispanic map. Map lengths are expected to vary from one dataset to another based on differences in sample sizes, pedigree structure, genotyping completeness, and marker heterozygosities.

The accuracy of map estimates can be measured by the standard errors and the 95% CIs. Because of the large sample size of the European data, the standard errors for our enhanced sex-averaged map are quite small and the 95% CI for a 10 cM map interval in Europeans is only approximately 2.4 cM long.

Our European and Chinese enhanced maps are the first population-specific genetic maps constructed using a meta analysis approach to combine maps constructed using separate marker set-specific datasets. The method that we adopted has efficiency comparable to that of joint analysis of pooled data [24]. In addition, combining maps from different datasets can avoid the practical difficulty of pooling a large heterogeneous data collection for a joint analysis. Without any need to access our original data, other investigators can easily incorporate their own data and improve these maps in the future.

The enhanced linkage maps from this study are being used to improve estimates of map distances on the Rutgers Map [42]. The Rutgers Map provide map positions for over 28,000 markers (SNPs and microsatellite markers) using a combination of physical positions and linkage-based distance estimates. The Rutgers Map interpolation tool can be used to interpolate linkage map positions for any marker based on its physical position. This resource facilitates the use of genetic maps of SNPs for genome scans for linkage to genetic traits. While the Rutgers Map includes nearly all markers available for construction of linkage maps, these markers were only genotyped in a relatively small pedigree set, with an average of 301 informative meioses per marker. Incorporation of the map distance estimates obtained from these enhanced linkage maps will improve the accuracy of the Rutgers Maps.

## Conclusion

In summary, we have evaluated 461 markers from the common Weber screening set maps using a very large set of genotype data. We used these data to obtain highly precise estimates of recombination-based map distances and to correct marker order discrepancies, resulting in enhanced linkage maps that can facilitate more accurate genome-wide linkage analyses. We also used these data to identify several discrepancies in map distances between specific ethnic populations, and to provide population-specific maps for African Americans, Chinese, Hispanic, and Palauan samples. For regions where map lengths differ among populations, using the population-specific map distances may allow for more accurate linkage analyses. Our data support the suggestion that there may be population differences in genomic structure, and that ignoring such differences could have a negative impact on genetic analyses.

## Authors' contributions

CH contributed to study design and performed all analyses; DEW and TCM designed, obtained funding for, and coordinated the project; SB advised on statistical analyses and discussed results; GA and WS provided specialized software; CH, TCM, DEW wrote the manuscript, and all other authors critically read the manuscript; the scientists in the Enhanced Genetic Map Consortium contributed all of the genotype data used for this project; All authors have read and approved the final manuscript.

## Pre-publication history

The pre-publication history for this paper can be accessed here:

http://www.biomedcentral.com/1471-2350/12/15/prepub

## Supplementary Material

Additional file 1 **Enhanced linkage maps for the European population**. Detailed enhanced linkage map in the European population. Click here for file

Additional file 2 **Enhanced linkage maps for the Chinese population**. Detailed enhanced linkage map in the Chinese population. Click here for file

Additional file 3 **Enhanced linkage maps for the African American population**. Detailed enhanced linkage map in the African American population. Click here for file

Additional file 4 **Enhanced linkage maps for the Hispanic population**. Detailed enhanced linkage map in the Hispanic population. Click here for file

Additional file 5 **Enhanced linkage maps for the Palauan population**. Detailed enhanced linkage map in the Palauan population. Click here for file

Additional file 6 **Map interval comparisons between populations**. Q-Q plots of the Z-scores for sex-averaged map interval differences between populations. In each comparison of population A vs. population B, a point lies above the red reference line if the map length in population A was longer than in population B. Click here for file

## References

[B1] Botstein D White RL Skolnick M Davis RW Construction of a genetic linkage map in man using restriction fragment length polymorphisms Am J Hum Genet 1980 32 3 314 331 PMC1686077 6247908

[B2] A comprehensive genetic linkage map of the human genome. NIH/CEPH Collaborative Mapping Group Science 1992 258 5079 148 162 1359639

[B3] Gyapay G Morissette J Vignal A Dib C Fizames C Millasseau P Marc S Bernardi G Lathrop M Weissenbach J The 1993-94 Genethon human genetic linkage map Nat Genet 1994 7 2 Spec No 246 339 10.1038/ng0694supp-246 7545953

[B4] Murray JC Buetow KH Weber JL Ludwigsen S Scherpbier-Heddema T Manion F Quillen J Sheffield VC Sunden S Duyk GM Weissenbach J Gyapay G Dib C Morrissette J Lathrop GM Vignal A White R Matsunami N Gerken S Melis R Albertsen H Plaetke R Odelberg S Ward D Dausset J Cohen D Cann H A comprehensive human linkage map with centimorgan density. Cooperative Human Linkage Center (CHLC) Science 1994 265 5181 2049 2054 10.1126/science.8091227 8091227

[B5] Matise TC Perlin M Chakravarti A Automated construction of genetic linkage maps using an expert system (MultiMap): a human genome linkage map Nat Genet 1994 6 4 384 390 10.1038/ng0494-384 8054979

[B6] Dib C Faure S Fizames C Samson D Drouot N Vignal A Millasseau P Marc S Hazan J Seboun E Lathrop M Gyapay G Morissette J Weissenbach J A comprehensive genetic map of the human genome based on 5,264 microsatellites Nature 1996 380 6570 152 154 10.1038/380152a0 8600387

[B7] Broman KW Murray JC Sheffield VC White RL Weber JL Comprehensive human genetic maps: individual and sex-specific variation in recombination Am J Hum Genet 1998 63 3 861 869 10.1086/302011 PMC1377399 9718341

[B8] Dausset J Cann H Cohen D Lathrop M Lalouel JM White R Centre d'etude du polymorphisme humain (CEPH): collaborative genetic mapping of the human genome Genomics 1990 6 3 575 577 10.1016/0888-7543(90)90491-c 2184120

[B9] Kong A Gudbjartsson DF Sainz J Jonsdottir GM Gudjonsson SA Richardsson B Sigurdardottir S Barnard J Hallbeck B Masson G Shlien A Palsson ST Frigge ML Thorgeirsson TE Gulcher JR Stefansson K A high-resolution recombination map of the human genome Nat Genet 2002 31 3 241 247 10.1038/ng917 12053178

[B10] Weber JL The Iceland map Nat Genet 2002 31 3 225 226 10.1038/ng920 12053179

[B11] Daw EW Thompson EA Wijsman EM Bias in multipoint linkage analysis arising from map misspecification Genet Epidemiol 2000 19 4 366 380 10.1002/1098-2272(200012)19:4<366::AID-GEPI8>3.0.CO;2-F 11108646

[B12] Fingerlin TE Abecasis GR Boehnke M Using sex-averaged genetic maps in multipoint linkage analysis when identity-by-descent status is incompletely known Genet Epidemiol 2006 30 5 384 396 10.1002/gepi.20151 16685713

[B13] Halpern J Whittemore AS Multipoint linkage analysis. A cautionary note Hum Hered 1999 49 4 194 196 10.1159/000022874 10436380

[B14] Yuan B Vaske D Weber JL Beck J Sheffield VC Improved set of short-tandem-repeat polymorphisms for screening the human genome Am J Hum Genet 1997 60 2 459 460 PMC1712401 9012420

[B15] Weitkamp LR Proceedings: Population differences in meiotic recombination frequency between loci on chromosome 1 Cytogenet Cell Genet 1974 13 1 179 182 10.1159/000130267 4208017

[B16] Jorgenson E Tang H Gadde M Province M Leppert M Kardia S Schork N Cooper R Rao DC Boerwinkle E Risch N Ethnicity and human genetic linkage maps Am J Hum Genet 2005 76 2 276 290 10.1086/427926 PMC1196373 15627237

[B17] Ju YS Park H Lee MK Kim JI Sung J Cho SI Seo JS A genome-wide Asian genetic map and ethnic comparison: the GENDISCAN study BMC Genomics 2008 9 554 10.1186/1471-2164-9-554 PMC2612022 19025666

[B18] McPeek MS Sun L Statistical tests for detection of misspecified relationships by use of genome-screen data Am J Hum Genet 2000 66 3 1076 1094 10.1086/302800 PMC1288143 10712219

[B19] O'Connell JR Weeks DE PedCheck: a program for identification of genotype incompatibilities in linkage analysis Am J Hum Genet 1998 63 1 259 266 10.1086/301904 PMC1377228 9634505

[B20] Gudbjartsson DF Jonasson K Frigge ML Kong A Allegro, a new computer program for multipoint linkage analysis Nat Genet 2000 25 1 12 13 10.1038/75514 10802644

[B21] Lander ES Green P Construction of multilocus genetic linkage maps in humans Proc Natl Acad Sci USA 1987 84 8 2363 2367 10.1073/pnas.84.8.2363 PMC304651 3470801

[B22] Lange K Cantor R Horvath S Perola M Sabatti C Sinsheimer J Sobel E MENDEL version 4.0: A complete package for the exact genetic analysis of discrete traits in pedigree and population data sets Am J Hum Genet 2001 69 supplement 504

[B23] Abecasis GR Cherny SS Cookson WO Cardon LR Merlin--rapid analysis of dense genetic maps using sparse gene flow trees Nat Genet 2002 30 1 97 101 10.1038/ng786 11731797

[B24] Stewart WC Improving estimates of genetic maps: a meta-analysis-based approach Genet Epidemiol 2007 31 5 408 416 10.1002/gepi.20221 17443710

[B25] DeWan AT Parrado AR Matise TC Leal SM The map problem: a comparison of genetic and sequence-based physical maps Am J Hum Genet 2002 70 1 101 107 10.1086/324774 PMC384881 11706388

[B26] Murphy K Raj T Winters RS White PS me-PCR: a refined ultrafast algorithm for identifying sequence-defined genomic elements Bioinformatics 2004 20 4 588 590 10.1093/bioinformatics/btg466 14990458

[B27] Schuler GD Sequence mapping by electronic PCR Genome Res 1997 7 5 541 550 10.1101/gr.7.5.541 PMC310656 9149949

[B28] Goldgar DE Green P Parry DM Mulvihill JJ Multipoint linkage analysis in neurofibromatosis type I: an international collaboration Am J Hum Genet 1989 44 1 6 12 PMC1715467 2491784

[B29] Stewart WC Thompson EA Improving estimates of genetic maps: a maximum likelihood approach Biometrics 2006 62 3 728 734 10.1111/j.1541-0420.2006.00532.x 16984314

[B30] Dempster A Laird N Rubin D Maximum likelihood from incomplete data via the EM algorithm J Roy Statist Soc 1977 39 1 38

[B31] Efron B Tibshirani R Statistical data analysis in the computer age Science 1991 253 5018 390 395 10.1126/science.253.5018.390 17746394

[B32] Byrd RH Lu P Nocedal J Zhu C A limited memory algorithm for bound constrained optimization SIAM J Scientific Computing 1995 16 1190 1208

[B33] Storey JD Tibshirani R Statistical significance for genomewide studies Proc Natl Acad Sci USA 2003 100 16 9440 9445 10.1073/pnas.1530509100 PMC170937 12883005

[B34] Benjamini Y Hochberg Y Controlling the false discovery rate: a practical and powerful approach to multiple testing JRSSB 1995 57 125 133

[B35] Clopper C Pearson E The use of confidence or fiducial limits illustrated in the case of the binomial Biometrika 1934 26 404 413

[B36] Broman K Matsumoto N Giglio S Martin C Roseberry J Zuffardi O Ledbetter D Weber J eds Common long human inversion polymorphism on chromosome 8p. Science and Statistics: A Festschrift for Terry Speed 2003

[B37] Cherny SS Abecasis GR Cookson WO Sham PC Cardon LR The effect of genotype and pedigree error on linkage analysis: analysis of three asthma genome scans Genet Epidemiol 2001 21 Suppl 1 S117 122 10.1002/gepi.2001.21.s1.s117 11793653

[B38] Lange K Weeks D Boehnke M Programs for Pedigree Analysis: MENDEL, FISHER, and dGENE Genet Epidemiol 1988 5 6 471 472 10.1002/gepi.1370050611 3061869

[B39] Giglio S Broman KW Matsumoto N Calvari V Gimelli G Neumann T Ohashi H Voullaire L Larizza D Giorda R Weber JL Ledbetter DH Zuffardi O Olfactory receptor-gene clusters, genomic-inversion polymorphisms, and common chromosome rearrangements Am J Hum Genet 2001 68 4 874 883 10.1086/319506 PMC1275641 11231899

[B40] Shimokawa O Kurosawa K Ida T Harada N Kondoh T Miyake N Yoshiura K Kishino T Ohta T Niikawa N Matsumoto N Molecular characterization of inv dup del(8p): analysis of five cases Am J Med Genet A 2004 128A(2): 133 137 10.1002/ajmg.a.30063 15214003

[B41] Tuzun E Sharp AJ Bailey JA Kaul R Morrison VA Pertz LM Haugen E Hayden H Albertson D Pinkel D Olson MV Eichler EE Fine-scale structural variation of the human genome Nat Genet 2005 37 7 727 732 10.1038/ng1562 15895083

[B42] Matise TC Chen F Chen W De La Vega FM Hansen M He C Hyland FC Kennedy GC Kong X Murray SS Ziegle JS Stewart WC Buyske S A second-generation combined linkage physical map of the human genome Genome Res 2007 17 12 1783 1786 10.1101/gr.7156307 PMC2099587 17989245

