# Supplementary material for: Enhanced genetic maps from family-based disease studies: population-specific comparisons
Source: BMC Med Genet. 2011 Jan 19;12:15. doi: 10.1186/1471-2350-12-15 (PMC3037840; doi:10.1186/1471-2350-12-15)
Supplement: Additional file 6 — Map interval comparisons between populations. Q-Q plots of the Z-scores for sex-averaged map interval differences between populations. In each comparison of population A vs. population B, a point lies above the red reference line if the map length in population A was longer than in population B. [file 1471-2350-12-15-S6.PDF]

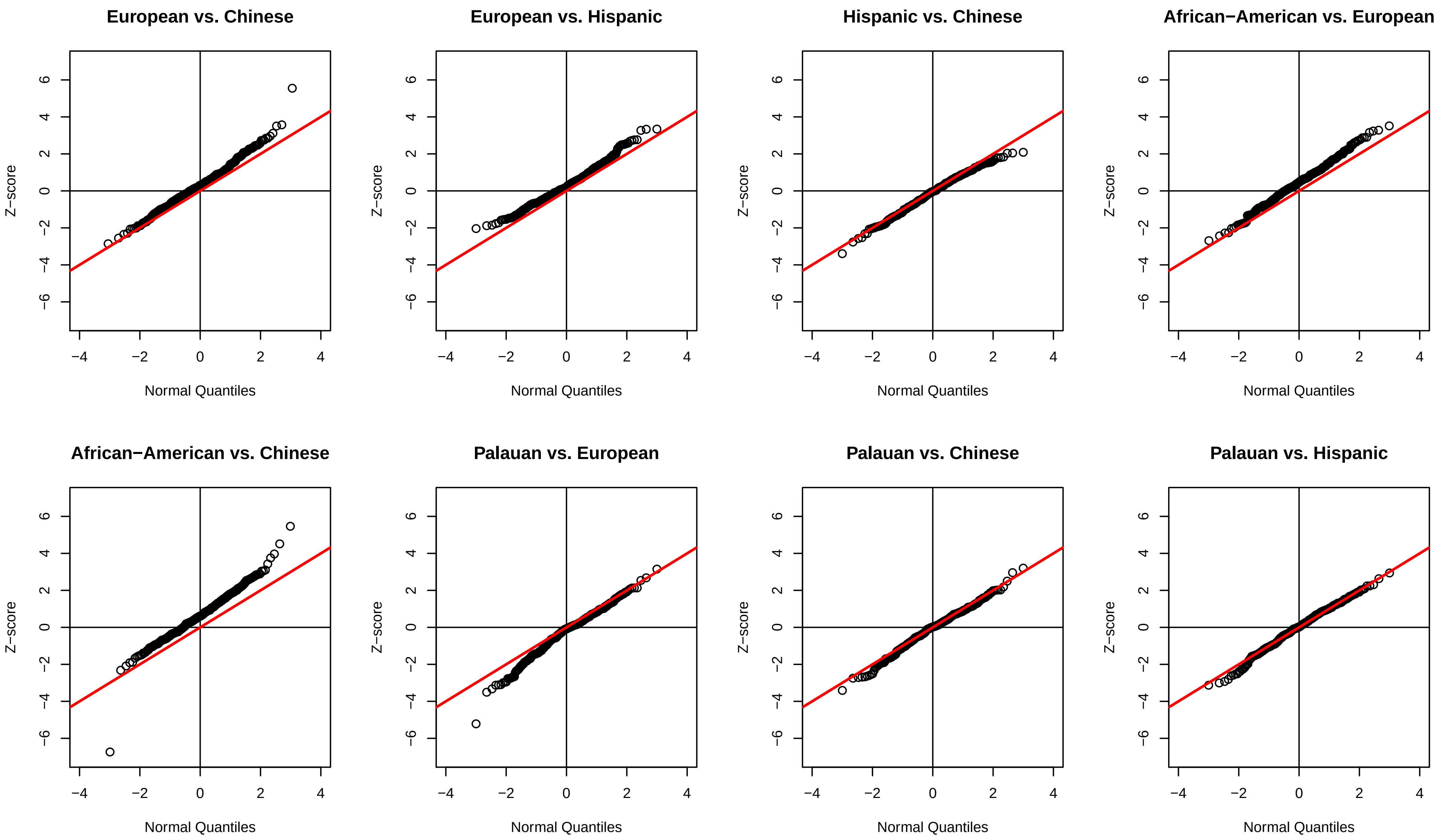

European vs. Chinese
European vs. Hispanic
Hispanic vs. Chinese
African−American vs. European
African−American vs. Chinese
Palauan vs. European
Palauan vs. Chinese
Palauan vs. Hispanic
Z−score
Normal Quantiles
